# SMARCA4 and SMARCE1 in gastric cancer: Correlation with ARID1A, and microsatellite stability, and *SMARCE1* / *ERBB2* co‐amplification

**DOI:** 10.1002/cam4.5776

**Published:** 2023-03-14

**Authors:** Katharina Pries, Sandra Krüger, Steffen Heckl, Hans‐Michael Behrens, Christoph Röcken

**Affiliations:** ^1^ Department of Pathology Christian‐Albrechts‐University, University‐Hospital Schleswig‐Holstein Kiel Germany; ^2^ Department of Internal Medicine II Christian‐Albrechts‐University, University‐Hospital Schleswig‐Holstein Kiel Germany

**Keywords:** gastric carcinoma, heterogeneity, immunohistochemistry, MSI, SMARCA4, SMARCE1

## Abstract

**Background:**

Recent studies have shown an association between certain subunits of the SWI/SNF complex with specific tumor characteristics in gastric cancer (GC). In an earlier study, we applied multiregional whole exome sequencing on multiple primary tumor samples and found alterations of the SWI/SNF complex in 78% of the cases. *ERBB2*, which encodes for Her2/neu, is a well‐known predictive biomarker used to guide the treatment of GC in the palliative setting. *SMARCE1*, which encodes for a subunit of the SWI/SNF complex, is localized in close genetic proximity to *ERBB2*.

**Aim:**

As little is known about the significance of the SWI/SNF complex in GC biology and the potential relationship between *ERBB2* and *SMARCE1* upregulation, we examined the expression patterns of SMARCA4 and SMARCE1, two mutually exclusive catalytic ATPase subunits of the SWI/SNF complex, in a well characterized GC cohort.

**Materials and Methods:**

The expression of SMARCA4 and SMARCE1 was studied by immunohistochemistry in connection with clinicopathological patient characteristics in a cohort of 468 GCs. Digital droplet polymerase chain reaction was performed for amplification analysis on *ERBB2* and *SMARCE1*.

**Results:**

Immunohistochemical staining of whole‐mount tissue sections found a diffusely “gray scale” expression of SMARCA4 in 446 (95.2%) GCs and of SMARCE1 in 463 (98.8%) GCs. The expression of SMARCA4 and SMARCE1 correlated significantly with ARID1A, p53, and microsatellite status. No correlation was found with the patient prognosis. The amplification analysis of *SMARCE1* showed amplification in 4 of 34 cases. In 3 of 34 cases, *SMARCE1* was co‐amplified with *ERBB2*. We also found a co‐expression of SMARCE1 and Her2/neu in a subset of patients.

**Conclusion:**

While the effect of a co‐amplification is currently unknown, synergistic effects of SMARCE1 and Her2/neu overexpression should be explored in future studies, holding potential for an improved treatment of GC.

## Graphical abstract

Expression of SMARCA4 and SMARCE1 in gastric cancer as illustrated by immunohistochemistry.

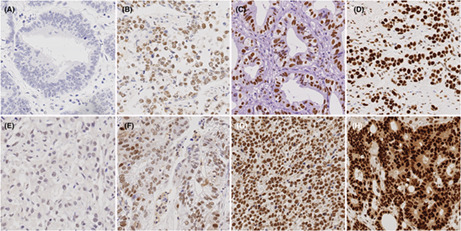

## 1 INTRODUCTION

The SWI/SNF complex plays a crucial role in epigenetic regulation by remodeling chromatin in an ATP‐dependent manner and is dysregulated in many different cancers, in general, and particularly in gastric cancer (GC), the third leading cause of cancer‐related deaths worldwide.^1^ SWI/SNF‐Related, Matrix‐Associated, Actin‐Dependent Regulator of Chromatin, Subfamily A, Member 4 (SMARCA4, synonymous: BRG1) is one of the two mutually exclusive catalytic ATPase subunits required for the nucleosome transitioning activity of the SWI/SNF complex.^2^ Loss‐of‐function mutations of some of the subunits are common events in cancer.^2^, ^3^ While loss‐of‐function mutations cause dysregulation of the complex, evidence indicates that upregulation of SWI/SNF‐Related, Matrix‐Associated, Actin‐Dependent Regulator of Chromatin, Subfamily E, Member 1 (SMARCE1, synonymous: BAF57) is associated with a poor prognosis, increased cell growth and metastasis in GC.^4^ AT‐rich Interaction Domain 1 A (ARID1A) is another subunit of the SWI/SNF complex, which is dysregulated in GC. Partial or complete loss of ARID1A in GC correlated significantly with tumor type according to Laurén, Epstein–Barr virus status, microsatellite instability, PD‐L1 status, and nodal spread,^5^ supporting the notion that the SWI/SNF complex contributes to GC biology. In a recent study, we applied multiregional whole exome sequencing on multiple primary tumor samples obtained from nine patients with an adenocarcinoma of the stomach or gastroesophageal junction. Alterations of the SWI/SNF complex were found in seven (78%) cases, including single nucleotide variations, deletions, and amplifications.^6^ Furthermore and unlike the other tested subunits of the SWI/SNF complex, SMARCA4 and SMARCE1 were homogeneously affected, that is, discovered in every tumor sample of a primary tumor each in two cases, respectively.^6^ Interestingly, *SMARCE1* is localized on chromosome 17 in close proximity to *ERBB2*, which encodes the Human Epidermal Growth Factor Receptor 2 (Her2/neu). Her2/neu is a predictive biomarker used to guide the treatment of GC with an anti‐Her2/neu antibody Trastuzumab in the palliative setting.^7^, ^8^ Currently, little is known about the significance of the SWI/SNF complex in GC biology and the potential relationship between *ERBB2* and *SMARCE1* upregulation.

To fill this gap of information, we studied the expression of SMARCA4 and SMARCE1 in a large and well‐characterized European cohort of GC patients. We aimed to test the following hypotheses: (1) SMARCA4 and SMARCE1 are differentially expressed in GC and show intratumoral heterogeneity; (2) The expression of SMARCA4 and SMARCE1 correlates with clinicopathological patient characteristics, including EBV, MSI, and ARID1A status, (3) *SMARCE1* is co‐amplificated with *HER2*.

## 2 MATERIALS AND METHODS

### 2.1 Ethics

All executed procedures were in accordance with the ethical standards of the responsible committee on human experimentation (institutional and national) and with the Helsinki Declaration of 1964 and later versions. Ethical approval was obtained from the local ethical review board (D 453/10 and D 525/15). All experimental work complied with all mandatory laboratory health and safety procedures.

### 2.2 Study population

From the archive of the Department of Pathology, University‐Hospital Schleswig‐Holstein, patients who had undergone either total or partial gastrectomy for adenocarcinoma of the stomach or esophago‐gastric‐junction between 1997 and 2009 were identified. The following patient characteristics were retrieved: type of surgery, age at diagnosis, gender, tumor size, tumor localization, tumor type, tumor grade, depth of invasion, residual tumor status, number of lymph nodes resected, and number of lymph nodes with metastases. Patients were included if histology confirmed adenocarcinoma of the stomach or esophago‐gastric‐junction. Patients were excluded if histology identified a tumor type other than adenocarcinoma, if patients had a partial resection of the stomach in which remnant GC occurred later on or if neoadjuvant/perioperative therapy was applied. Date of patient death was obtained from the Epidemiological Cancer Registry of the state of Schleswig‐Holstein, Germany. Follow‐up data of those patients who were still alive were retrieved from hospital records and general practitioners. All patient data were pseudonymized after study inclusion.

### 2.3 Histology

Tissue specimens were fixed in formalin and embedded in paraffin (FFPE). Deparaffinized sections were stained with hematoxylin and eosin. Tumors were classified according to Laurén.^9^ pTNM stage of all study patients was determined according to the eighth edition of the UICC guidelines.^10^

### 2.4 Assessment of microsatellite instability and detection of *Helicobacter pylori* and Epstein–Barr virus infection

The *H*. *pylori*, microsatellite instability, Epstein–Barr virus, HER2, MET, *PIK3CA*, PD‐L1, PD‐1, E‐cadherin, p53, and ARID1A status was assessed as described in detailed elsewhere.^5^, ^11^, ^12^, ^13^, ^14^, ^15^, ^16^

### 2.5 Immunohistochemical detection of SMARCA4 and SMARCE1

Immunohistochemical staining was carried out using the Bond‐Max (Leica Biosystems), an automated slide staining system. Pretreatment was done with ER2 (Leica Biosystems) for 20 min for both SMARCA4 and SMARCE1. A monoclonal mouse antibody against SMARCA4/ BRG1 (clone E8V5B, Cell Signaling Technology) applied in a 1:400 dilution and a monoclonal rabbit antibody, directed against SMARCE1/BAF57 (clone E6H5J, Cell Signaling Technology) applied in a 1:300 dilution were used. Immunostaining was visualized with the Polymer Refine Detection Kit (Leica Biosystems). Hemalaun served as counterstaining.

### 2.6 Evaluation of SMARCA4 and SMARCE1 immunostaining

**FIGURE 1:**
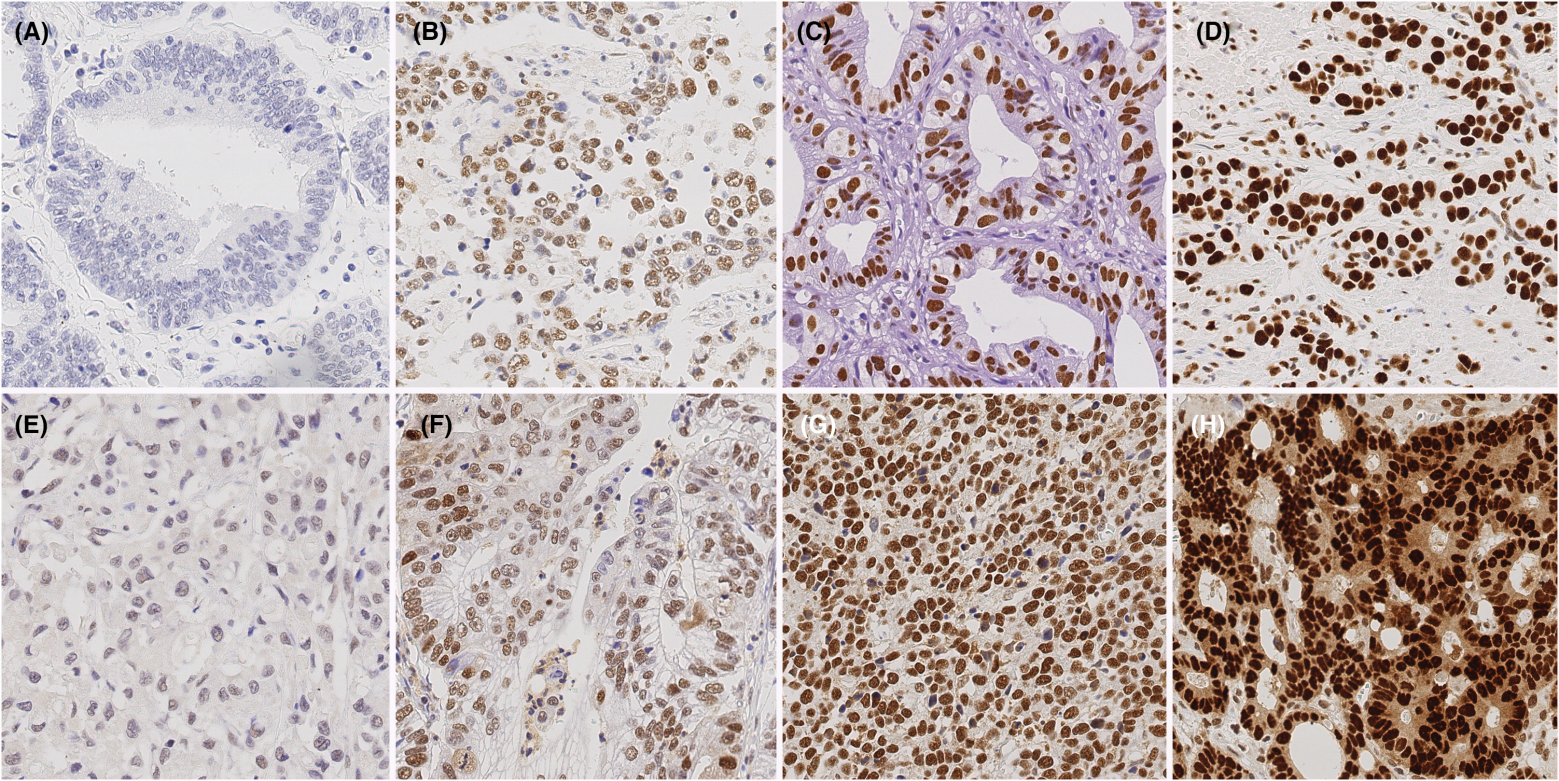
References for immunostaining analysis according to H‐score. Staining intensities ranged from 0 (A/E; negative) to 3+ (D/H, strong expression) with 1+ (B/F; weak expression) and 2+ (C/G; moderate expression) in between. Lymphocytes served as internal positive control. Upper row: Anti‐SMARCA4 immunostaining, hematoxylin counterstain; original magnification ×400; lower row: Anti‐SMARCE1 immunostaining, hematoxylin counterstain; original magnification ×400.

Nuclear expression of SMARCA4 and SMARCE1 was evaluated using the histoscore (H‐score): The first parameter was based on the intensity of the stained tumor cells. A score of 0 (no staining), 1+ (weak), 2+ (moderate), and 3+ (strong staining reaction) was applied (Figure 1). The second parameter estimated the distribution of the stained tumor cells in percentage. The total of all staining intensities found in a single case are always added to a total of 100% according to the following formula: % (0) + % (1+) + % (2+) + % (3+) = 100%. Finally, the H‐score was calculated to the following formula: 0 × (% of immunonegative tumor cells) + 1 × (% of weakly stained tumor cells) + 2 × (% of moderately stained tumor cells) + 3 × (% of strongly stained tumor cells) = H‐score. The H‐score ranged from 0 [=0 × (100% immunonegative tumor cells)] to 300 [=3 × (100% of strongly stained tumor cells)]. Four representative cases in the intensities 0–3+ were used as a reference standard for the evaluation of the entire cohort (Figure 1).

### 2.7 Comparing the expression of SMARCE1 and HER2

Out of the 468 included cases 36 had been classified as HER2 positive.^14^ One case was excluded because the HER2‐positive area was missing in the deeper tissue sections stained with the anti‐SMARCE1 antibody. Examining the concordance of the SMARCE1 positive and HER2 positive tumor areas, the immunohistochemical SMARCE1 stained tissue sections were compared with Her2/neu immunohistochemical and chromogenic in situ hybridization stained tissue sections from a previous study using a microscope and digitalized scans of the stained slides.^14^ The cases were assigned into the following groups: complete concordance, partial concordance, and no concordance.

### 2.8 Amplification analysis of SMARCE1

The amplification of *SMARCE1* was studied by droplet digital polymerase chain reaction (ddPCR). Out of the 468 included cases we selected 34 cases for the amplification analysis. 18 of these cases, which all presented a low H‐score, indicating a low expression, served as a negative control. 16 cases of the 34 selected cases showed a high H‐score, indicating a high expression, therefore serving as potential amplified cases. For each case, DNA was extracted from FFPE tissue using the DNA mini kit (Qiagen). SMARCE1 primers and probes were self‐designed and custom‐made by Biomers as follows: forward: 5′‐ACACTCCTTTTTATGGTTACTGGT‐3′ and reverse: 5′‐TTCTCTGGAAACGGGCGGT‐3′, Probe: 5′‐6‐FAM‐TGTTCACTCCAGATTATGATGATGGC‐BMN‐Q530‐3′; EFTUD2 Sequences were taken as reference control from Liu et al.^4^ and used as follows: forward 5′‐GGTCTTGCCAGACACCAAAG‐3′ and reverse 5′‐TGAGAGGACACACGCAAAAC‐3′, Probe 5′‐6‐HEX‐GGACATCCTTTGGCTTTTGA‐BMN‐Q530‐3′. The ddPCR was performed using the ddPCR Supermix for Probes (No dUTP) (Bio‐Rad), the QX200 Droplet Generator (Bio‐Rad), C100 Touch Thermocycler with DeepWell Reaction Module (Bio‐Rad), and a QX Droplet Reader (Bio‐Rad). To analyze the ddPCR results QuantaSoft™ Software version 1.7 (Bio‐Rad) was used.

### 2.9 Statistical analysis

Statistical analyses were done using SPSS 27.0 (IBM Corporation). First, the H‐score was dichotomized at the median (≤175 H‐score vs. >175 H‐score for SMARCA4 and <120 H‐score vs. ≥120 H‐score for SMARCE1), separating the data into groups with low/negative and moderate/high expression of SMARCA4 and SMARCE1, respectively. In order to test our hypotheses, we then performed subgroup analyses of the first quartile (<111 H‐score) versus the other three quartiles (≥111 H‐score) for SMARCA4 and the first three quartiles (≤170 H‐score) versus the fourth quartile (>170 H‐score) for SMARCE1. The significance of the correlation between the subgroups and clinicopathological characteristics was calculated using the Fisher's exact test. For parameters of ordinal scale (T‐category, N‐category, UICC stage), we applied the Kendall's tau test additionally. We assumed a significance level of 0.05. To compensate false discovery rate within the correlation we applied the Simes (Benjamini–Hochberg) procedure (multiple testing correction). All *p*‐values are uncorrected. The *p*‐values that remained significant after FDR correction are indicated. Median overall survival (OS) and tumor‐specific survival (TSS) were determined using the Kaplan–Meier method; therefore, the log‐rank test was used to determine the significance of difference.

## 3 RESULTS

In total, 468 patients fulfilled all study criteria. The clinicopathological patient characteristics are summarized in Table 1. According to Laurén, an intestinal phenotype was found in 240 (51.3%), a diffuse type in 148 (31.6%), a mixed type in 31 (6.6%), and an unclassifiable type in 49 (10.5%) patients. OS data were available in 455 cases (97.2%) for SMARCA4 and 456 cases (97.4%) for SMARCE1. TSS data were available in 426 cases (91.0%) for SMARCA4 and 427 cases (91.2%) for SMARCE1. The median follow‐up period was 30.8 months (range 0–129.9 months; Table 1).

**TABLE 1:** Correlation of nuclear SMARCA4 and SMARCE1 expression with clinicopathological patient characteristics.

			Total		SMARCA4 nuclear expression				SMARCE1 nuclear expression			
					H‐score low /negative (≤175)		H‐score high/positive (>175)		H‐score low/negative (<120)		H‐score high/positive (≥120)	
			*n*	(%)	*n*	(%)	*n*	(%)	*n*	(%)	*n*	(%)
Total												
					236	(50.4)	231	(49.4)	232	(49.6)	236	(50.4)
Gender	*n*	*p* ^a^			467			0.128	468			0.704
Female			179	(38.2)	82	(45.8)	97	(54.2)	91	(50.8)	88	(49.2)
Male			289	(61.8)	154	(53.5)	134	(46.5)	141	(48.8)	148	(51.2)
Age group	*n*	*p* ^a^			467			0.267	468			0.355
<68 years			237	(50.6)	113	(47.9)	123	(52.1)	112	(47.3)	125	(52.7)
≥68 years			231	(49.4)	123	(53.2)	108	(46.8)	120	(51.9)	111	(48.1)
Localization	*n*	*p* ^a^			465			0.369	466			0.046
Proximal stomach			146	(31.3)	79	(54.1)	67	(45.9)	62	(42.5)	84	(57.5)
Distal stomach			320	(68.7)	157	(49.2)	162	(50.8)	169	(52.8)	151	(47.2)
Laurén phenotype	*n*	*p* ^a^			467			0.031	468			0.884
Intestinal			240	(51.3)	134	(56.1)	105	(43.9)	118	(49.2)	122	(50.8)
Diffuse			148	(31.6)	63	(42.6)	85	(57.4)	72	(48.6)	76	(51.4)
Mixed			31	(6.6)	12	(38.7)	19	(61.3)	15	(48.4)	16	(51.6)
Unclassifiable			49	(10.5)	27	(55.1)	22	(44.9)	27	(55.1)	22	(44.9)
Grading	*n*	*p* ^a^			467			0.021	468			0.588
G1/G2			110	(23.5)	66	(60.6)	43	(39.4)	52	(47.3)	58	(52.7)
G3/G4			358	(76.5)	170	(47.5)	188	(52.5)	180	(50.3)	178	(49.7)
T‐category	*n*	*p* ^b^			467			0.434	468			0.522
pT1a/pT1b			58	(12.4)	25	(43.9)	32	(56.1)	27	(46.6)	31	(53.4)
pT2			53	(11.3)	34	(64.2)	19	(35.8)	30	(56.6)	23	(43.4)
pT3			185	(39.5)	95	(51.4)	90	(48.6)	84	(45.4)	101	(54.6)
pT4a/pT4b			172	(36.8)	82	(47.7)	90	(52.3)	91	(52.9)	81	(47.1)
N‐category	*n*	*p* ^b^			466			0.153	467			0.949
pN0			134	(28.6)	76	(56.7)	58	(43.3)	68	(50.7)	66	(49.3)
pN1			64	(13.7)	28	(43.8)	36	(56.3)	28	(43.8)	36	(56.3)
pN2			84	(18.0)	45	(54.2)	38	(45.8)	45	(53.6)	39	(46.4)
pN3a/b			185	(39.6)	87	(47.0)	98	(53.0)	91	(49.2)	94	(80.8)
Lymph node ratio	*n*	*p* ^a^			466			0.518	467			0.926
Low (<0.189)			227	(48.6)	118	(52.2)	108	(47.8)	112	(49.3)	115	(50.7)
High (≥0.189)			240	(51.4)	118	(49.2)	122	(50.8)	120	(50.0)	10	(50.0)
M‐category	*n*	*p* ^a^			467			0.348	468			0.241
pM0			378	(80.8)	195	(51.7)	182	(48.3)	182	(48.1)	196	(51.9)
pM1			90	(19.2)	41	(45.6)	49	(54.4)	50	(55.6)	40	(44.4)
UICC stage	*n*	*p* ^b^			466			0.208	467			0.450
IA/IB			81	(17.3)	41	(50.6)	40	(49.4)	41	(50.6)	40	(49.4)
IIA/IIB			99	(21.2)	57	(58.2)	41	(41.8)	46	(46.5)	53	(53.5)
IIIA/IIIB/IIIC			197	(42.2)	97	(49.2)	100	(50.8)	95	(48.2)	102	(51.8)
IV			90	(19.3)	41	(45.6)	49	(54.4)	50	(55.6)	40	(44.4)
L‐category	*n*	*p* ^a^			448			0.158	449			0.131
pL0			219	(48.8)	118	(54.1)	100	(45.9)	115	(52.5)	104	(47.5)
pL1			230	(51.2)	109	(47.4)	121	(52.6)	104	(45.2)	126	(54.8)
V‐category	*n*	*p* ^a^			447			1.000	448			0.290
pV0			399	(89.1)	201	(50.5)	197	(49.5)	198	(49.6)	201	(50.4)
pV1			49	(10.9)	25	(51.0)	24	(49.0)	20	(40.8)	29	(59.2)
R status	*n*	*p* ^a^			463			0.575	464			0.575
pR0			406	(87.5)	208	(51.4)	197	(48.6)	198	(48.8)	208	(51.2)
pR1/pR2			58	(12.5)	27	(46.6)	31	(53.4)	31	(53.4)	27	(46.6)
HER2 status	*n*	*p* ^a^			436			0.487	437			0.223
Negative			401	(91.8)	205	(51.2)	195	(48.8)	204	(50.9)	197	(49.1)
Positive			36	(8.2)	21	(58.3)	15	(41.7)	14	(38.9)	22	(61.1)
SMARCE1 H‐score median	*n*	*p* ^a^			467			0.000*				
Low H‐score <120			232	(49.6)	148	(63.8)	84	(36.2)				
High H‐score ≥120			236	(50.4)	88	(37.4)	147	(62.6)				
ARID1A any expression versus complete loss	*n*	*p* ^a^			414			0.001*	415			0.000*
H‐score = 0 (Complete loss)			45	(10.8)	33	(73.3)	12	(26.7)	38	(84.4)	7	(15.6)
H‐score >0			370	(89.2)	170	(46.1)	199	(53.9)	157	(42.4)	213	(57.6)
ARID1A complete loss or B/W versus rest	*n*	*p* ^a^			436			0.003*	437			0.000*
Rest			370	(84.7)	170	(46.1)	199	(53.9)	157	(42.4)	213	(57.6)
Complete loss or B/W			67	(15.3)	44	(65.7)	23	(34.3)	52	(77.6)	15	(22.4)
TP53 H‐score median	*n*	*p* ^a^			458			0.191	459			0.007
Low (H‐score ≤91.5)			230	(50.1)	123	(53.5)	107	(46.5)	128	(55.7)	102	(44.3)
High (H‐score >91.5)			229	(49.9)	107	(46.9)	121	(53.1)	98	(42.8)	131	(57.2)
TP53 mutation	*n*	*p* ^a^			107			0.410	107			0.153
Wildtype or silent mutation			72	(67.3)	36	(50.0)	36	(50.0)	40	(55.6)	32	(44.4)
Mutation			35	(32.7)	14	(40.0)	21	(60.0)	14	(40.0)	21	(60.0)
SMAD4 Cyt H‐score Q123/Q4	*n*	*p* ^a^			457			0.419	458			0.050
Q123			364	(79.5)	188	(51.8)	175	(48.2)	189	(51.9)	175	(48.1)
Q4			94	(20.5)	44	(46.8)	50	(53.2)	38	(40.4)	56	(59.6)
SMAD4 Nuc H‐score 0	*n*	*p* ^a^			457			0.510	458			0.707
Present			260	(56.8)	135	(52.1)	124	(47.9)	131	(50.4)	129	(49.6)
Absent			198	(43.2)	97	(49.0)	101	(51.0)	96	(48.5)	102	(51.5)
*Helicobacter pylori*	*n*	*p* ^a^			395			0.889	396			0.674
Negative			336	(84.8)	173	(51.6)	162	(48.4)	170	(50.6)	166	(49.4)
Positive			60	(15.2)	30	(50.0)	30	(50.0)	28	(46.7)	32	(53.3)
EBV status	*n*	*p* ^a^			452			0.641	453			0.036
Negative			434	(95.8)	217	(50.1)	216	(49.9)	210	(48.4)	224	(51.6)
Positive			19	(4.2)	11	(57.9)	8	(42.1)	14	(73.7)	5	(26.3)
MSI status	*n*	*p* ^a^			451			0.004*	452			0.000*
MSS			417	(92.3)	203	(48.7)	214	(51.3)	194	(46.5)	223	(53.5)
MSI			35	(7.7)	26	(74.3)	9	(25.7)	30	(85.7)	5	(14.3)
E‐Cadherin	*n*	*p* ^a^			431			0.447	431			0.127
Negative			317	(73.5)	159	(50.2)	158	(49.8)	166	(52.4)	151	(47.6)
Positive			114	(26.5)	62	(54.4)	52	(45.6)	50	(43.9)	64	(56.1)
ß‐Catenin	*n*	*p* ^a^			433			0.629	433			0.122
Negative			245	(56.6)	126	(51.4)	119	(48.6)	128	(52.2)	117	(47.8)
Positive			188	(43.4)	92	(48.9)	96	(51.1)	84	(44.7)	104	(55.3)
Overall survival (months)	*p* ^c^				455			0.117	456			0.931
Total/events/censored					231/175/56		224/179/45		224/173/51		232/181/51	
Median survival					16.8 ± 2.1		13.4 ± 1.1		14.0 ± 1.8		15.6 ± 1.4	
95% CI					12.7–20.9		11.3–15.6		10.5–17.5		12.9–18.3	
Tumor‐specific survival (months)	*p* ^c^				426			0.383	427			0.610
Total/events/censored					216/146/70		210/143/67		207/142/65		220/147/73	
Median survival					18.4 ± 2.9		14.7 ± 1.4		14.7 ± 1.9		17.1 ± 1.9	
95% CI					12.7–24.2		12.0–17.4		11.0–18.3		13.3–20.8	

^a^ Fisher's exact test.

^b^ Kendall's tau test.

^c^ Log‐rank test.

* Significant after multiple testing corrections.

For 453 GCs, the EBV status was available with 19 (4.2%) EBV‐positive GCs. MSI status was available for 452 GCs with 35 (7.7%) positive GCs. MSI and EBV were mutually exclusive. 36 (8.2%) GCs were classified as HER2‐positive in tumor cells and 45 (10.8%) GCs showed a complete loss of ARID1A.

### 3.1 SMARCA4 and SMARCE1 immunostaining

Nuclear expression of SMARCE1 was found in tumor cells, non‐neoplastic and metaplastic epithelium, and most prominently and consistently in lymphocytes, which served as an internal positive control. The same, except for the expression in lymphocytes, was applied to the nuclear expression of SMARCA4. Staining intensities ranged from 0–3 with intensities 0, 1+, 2+ and 3+ found in 50.1%, 87.8%, 89.3%, and 53.1% of all cases for SMARCA4, and intensities 0, 1+, 2+, and 3+ found in 66.9%, 97.2%, 76.9% and 17.9% of all cases for SMARCE1, respectively (Figure 1). The percentage area of the four immunostaining categories (0–3+) for SMARCA4 ranged from 0% to 100% for category 0 and from 0% to 95% for weakly (1+), moderately (2+), and strongly (3+) stained areas. The percentage area of the four immunostaining categories (0–3+) for SMARCE1 ranged from 0% to 100% for category 0, from 0% to 99% for weakly stained areas (1+), from 0% to 95% for moderately stained areas (2+), and from 0% to 90% for strongly stained areas (3+). For SMARCA4 and SMARCE1 a single case showed no evidence of stained tumor cells at all (0 × 100% = H‐score 0).

### 3.2 SMARCA4 and SMARCE1 expression patterns

466 cases that were stained with the SMARCA4 antibody showed a combination of two or more staining intensities, that is, 0/1+ [43 cases (9.2%)], 0/2+ [4 cases (0.9%)], 1/ 2+ [66 cases (14.1%)], 2+/3+ [38 cases (8.1%)], 0/1+/2+ [101 cases (21.6%)], 0/1+/3+ [2 cases (0.4%)], 0/2+/3+ [10 cases (2.1%)], 1+/2+/3+ [129 cases (27.6%)], 0/1+/2+/3+ [69 cases (14.7%)].

467 cases that were stained with the SMARCE1 antibody showed a combination of two or more staining intensities, that is, 0/1+ [106 cases (22.6%)], 0/2+ [1 case (0.2%)], 1+/2+ [95 cases (20.3%)], 1+/3+ [1 case (0.2%)], 2+/3+ [9 cases (1.9%)], 0/1+/2+ [181 cases (38.7%)], 0/2+/3+ [2 cases (0.4%)] 1+/2+/3+ [50 cases (10.7%)], 0/1+/2+/3+ [22 cases (4.7%)].

**FIGURE 2:**
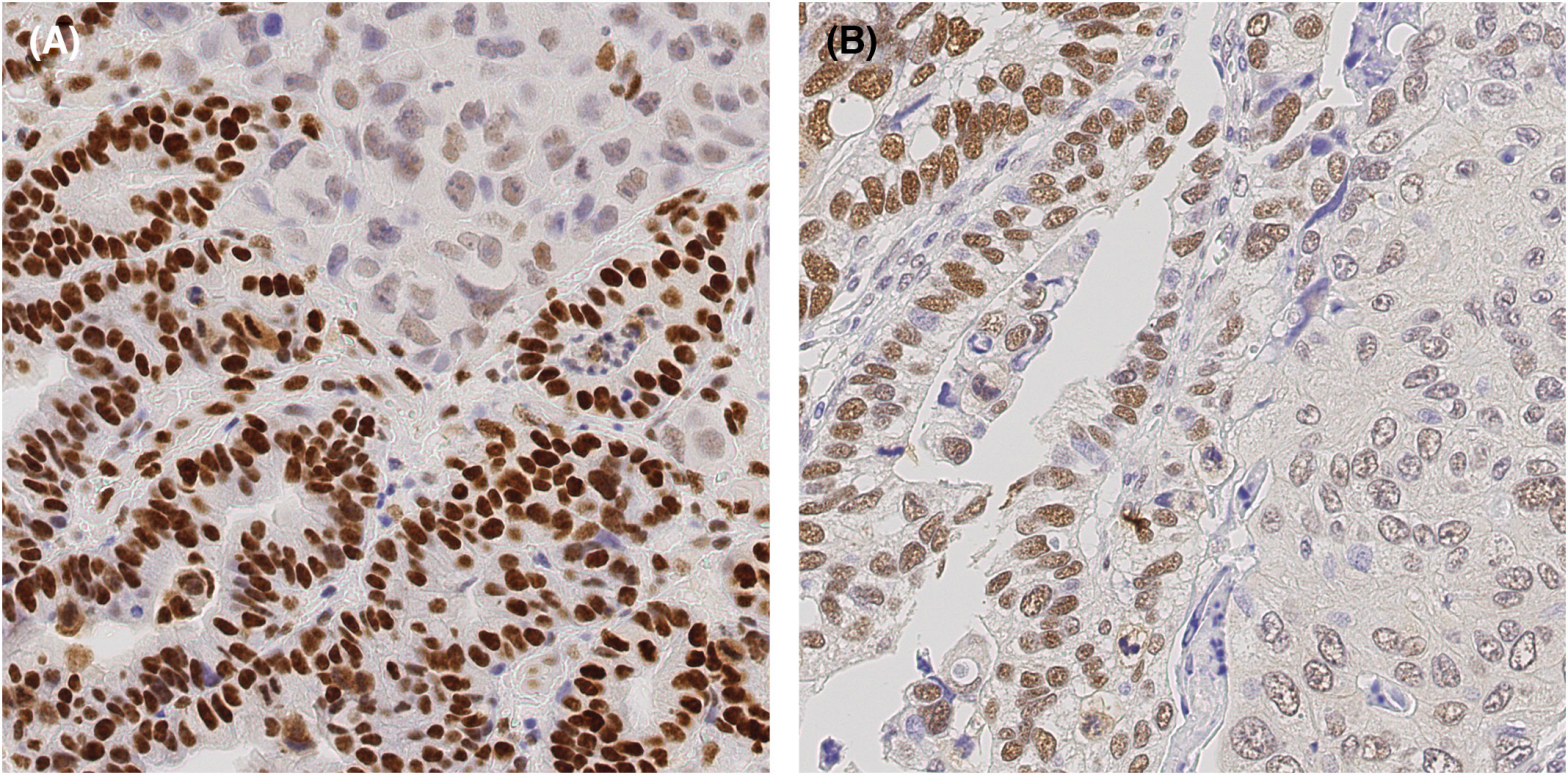
Patterns of heterogeneous SMARCA4 and SMARCE1 expression. While most cases depicted diffusely mixed heterogeneity in the expression of SMARCA4 and SMARCE1 (B), which we call a “gray scale” expression pattern, some cases showed distinctly separate areas of positive and negative expression as shown in (A) for SMARCA4, which we call a “black‐and‐white” expression pattern. Anti‐SMARCE1 immunostaining, hematoxylin counterstain; original magnification ×400. Anti‐SMARCA4 immunostaining, hematoxylin counterstain; original magnification ×400.

These data show that the expression (= combination of the intensity of immunostaining and amount of immunopositive tumor areas) of SMARCA4 and SMARCE1 was heterogeneous in GC, including “gray scale” and “black‐and‐white” immunostaining patterns (Figure 2).

### 3.3 Correlation with clinicopathological patient characteristics

To examine the potential biological significance of SMARCA4 and SMARCE1 in GC, we then correlated the expression patterns with various clinicopathological patient characteristics (Table 1). However, in view of a heterogenous expression and since we did not know a priori, which “cutoff” value of SMARCA4/SMARCE1 expression might be biologically relevant, we applied a stepwise explorative approach:

First, we correlated SMARCA4 and SMARCE1 expression according to the H‐score dichotomized at the medians with the clinicopathological patient characteristics (Table 1). Except for the microsatellite status, and ARID1A category, no correlation was found with any other parameter for SMARCA4. For SMARCE1 except for the microsatellite status, p53, and ARID1A category, no correlation was found with any other parameter (Table 1).

Second, we split the cohort into quartiles: for SMARCA4 Q1 (H‐score <111), Q2 (H‐score ≥111–≤175), Q3 (H‐score >175–<200), and Q4 (H‐score ≥200), and for SMARCE1 Q1 (H‐score ≤80), Q2 (H‐score >80–<120), Q3 (H‐score ≥120–≤170), and Q4 (H‐score >170). We then correlated the first quartile (<111 H‐score) versus the other three quartiles (≥111 H‐score) for SMARCA4 and the fourth quartile (>170 H‐score) versus the first three quartiles (≤170 H‐score) for SMARCE1 with the clinicopathological patient characteristics, in order to test a possible oncogenic influence of SMARCE1 and a potential tumor‐suppressing function of SMARCA4. Following this categorization SMARCA4 correlated significantly with the microsatellite, p53, and ARID1A status.

SMARCE1 correlated significantly with the microsatellite and ARID1A status.

Then, we correlated the expression of SMARCA4 with the expression of SMARCE1 to explore the significance of the subunit interaction of the SWI/SNF complex. Low expression of SMARCA4 significantly correlated with low expression of SMARCE1 and high expression of SMARCA4 correlated with high expression of SMARCE1 (*p* < 0.001, Table S1).

Finally, we split the cohort into the group “dysregulated SWI/SNF complex” versus “non dysregulated SWI/SNF complex.” The group “dysregulated SWI/SNF complex” included all cases in which either both H‐scores regarding SMARCA4 and SMARCE1 were identified as “low” (SMARCA4 H‐score <175; SMARCE1 H‐score <120) or “high” (SMARCA4 ≥175; SMARCE1 H‐score ≥120). Following this categorization, the group “dysregulated SWI/SNF complex” correlated significantly with the expression of ARID1A “any expression versus complete loss,” “complete loss or B/W versus rest” (*p* < 0.001, Table S1), the p53 H‐score (*p* = 0.01, Table S1), and the microsatellite status (*p* < 0.001, Table S1).

### 3.4 Concordance of the expression of SMARCE1 and HER2

**FIGURE 3:**
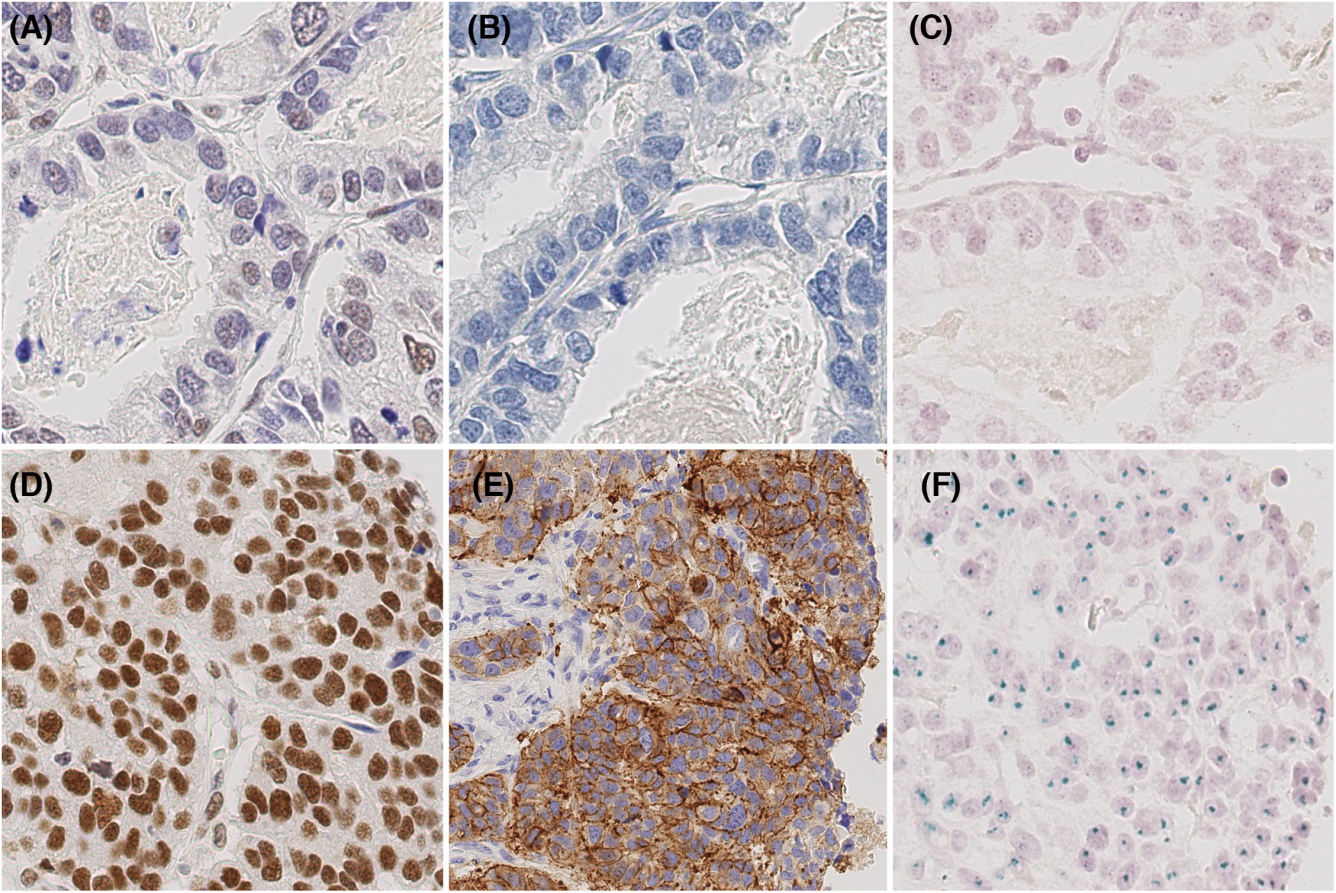
Comparison of the expression and amplification of SMARCE1 and HER2. Upper row: No expression of SMARCE1 (A) and HER2 (B) or amplification of HER2 (C) in the same area. Lower row: strong expression of SMARCE1 (D) and HER2 (E) and amplification of HER2 (F) in a different area of the same tissue as depicted in the upper row. Anti‐SMARCE1 immunostaining, hematoxylin counterstain; original magnification ×400. Anti‐HER2 immunostaining, hematoxylin counterstain; original magnification ×400. Detection of HER2 amplification with CISH, nuclear blue counterstain, original magnification ×400.

The comparison of the immunohistochemical stainings of SMARCE1 and Her2/neu showed a complete concordance of the SMARCE1 and HER2 positive areas, which as well matched with the HER2 positive areas in the chromogenic in situ hybridization of *HER2* in 20/35 cases. Only seven cases showed no concordance regarding the positive areas (Table S2, Figure 3).

### 3.5 Amplification of SMARCE1

The amplification analysis of *SMARCE1* showed an amplification of *SMARCE1* in 4 of 34 cases (CNV >3 absolute copies of *SMARCE1*, Table S2). Collectively, these data indicate that the expression of SMARCE1 is not only regulated by an amplification of *SMARCE1* in GC.

### 3.6 Amplification of SMARCE1 and ERBB2

Next, we applied ddPCR to study the amplification of *SMARCE1* in 34 cases with variable SMARCE1 expression with (*n* = 4) and without (*n* = 30) *ERBB2* amplification. Selection was based on the availability of sufficient amounts of FFPE tissue samples. *SMARCE1* amplification was found in 4 cases, of which 3 (75%) also showed *ERBB2* amplification (CNV >3 absolute copies of *SMARCE1*, Table S2) leading to the conjecture that *SMARCE1* and *ERBB2* can be co‐amplified in GC. In a single case, *ERBB2* amplification was present without the amplification of *SMARCE1* (Table S2).

### 3.7 Univariate survival analysis

**FIGURE 4:**
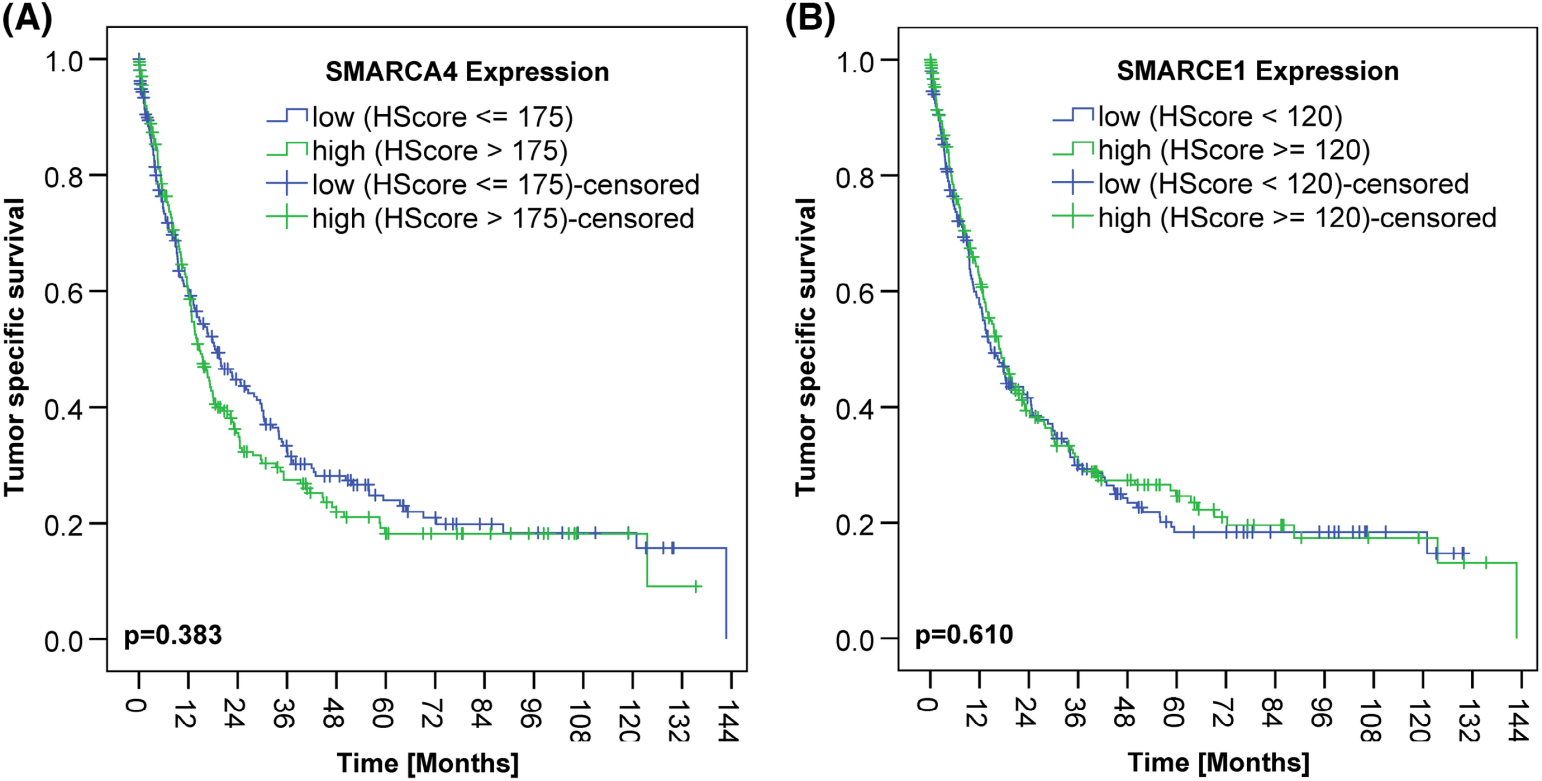
Kaplan–Meier curves for tumor‐specific survival (TSS). (A) SMARCA4 expression according to H‐score dichotomized by median showed no correlation with patient survival. (B) SMARCE1 expression according to H‐score dichotomized by median showed no correlation with patient survival.

Tumor‐specific and OS of patients depended significantly on several clinicopathological parameters and some biomarkers, that is, Laurén phenotype, T‐, M‐, N‐, V‐, and L‐category, lymph node ratio, UICC stage, tumor grade, as well as R, microsatellite, and PD‐L1 status. However, SMARCA4 and SMARCE1 expression according to H‐score dichotomized by median showed no correlation with patient survival, respectively (Table 1; Figure 4). Likewise, no correlations were found with patient survival when H‐scores were dichotomized at <111 versus ≥111 for SMARCA4 or ≤170 versus >170 for SMARCE1 (data not shown).

## 4 DISCUSSION

The SWI/SNF complex as a chromatin remodeler is a mediator of epigenetic modification and thus offers new possibilities for future precision medicine in the diagnostics and treatment of cancers. Yet the potential influence of a dysregulation of this complex on GC is largely unknown. Recently, a study has shown that a high expression of SMARCE1 leads to a poor prognosis and promotes metastasis in GC.^4^ For SMARCA4 different studies show inconsistent results, some state that an increased expression is associated with an advanced stage or poor prognosis,^18^, ^19^ while others indicate a tumor‐suppressor function of SMARCA4 with loss‐of‐function mutations being common in cancer.^2^, ^3^ This study of an independent White GC cohort aimed to examine the potential tumor biological significance regarding the expression of SMARCA4 and SMARCE1 along with the expression patterns and correlation with other important genetic characteristics like MSI and EBV status.

### 4.1 SMARCA4 and SMARCE1 are expressed heterogeneously in gastric cancer

Intratumoral heterogeneity is an often‐observed phenomenon for several biomarkers in GC.^11^, ^14^, ^20^, ^21^ Most of the examined cases showed a diffusely distributed expression pattern of different staining intensities within the same tumor for both SMARCA4 and SMARCE1. This so‐called “gray scale” was found in 95.2% (446 cases) of the SMARCA4‐stained GCs and 98.9% (463) of the SMARCE1‐stained GCs. Only one case showed a complete loss of SMARCE1 expression and five cases showed the same for SMARCA4, while three cases contained at least a partial loss (black/white pattern) of SMARCE1 and even 16 cases for SMARCA4. These findings indicate that the assessment of SMARCA4 and SMARCE1 loss in small tissue specimens, for example, biopsies, is susceptible to sampling errors.

### 4.2 SMARCA4 expression correlates with SMARCE1 expression

SMARCA4 and SMARCE1 are both subunits of the SWI/SNF complex.^2^ Our data show a correlation between the expression of SMARCA4 with SMARCE1 and vice versa. A low expression of SMARCA4 correlates significantly (*p* < 0.001, Table S1) with a low expression of SMARCE1 and a high expression of SMARCA4 is associated with a high expression of SMARCE1. Still, this does not allow any conclusion to be drawn about the dysregulation status of the subunits, as for SMARCE1 a high expression is classified as dysregulation while for SMARCA4 a loss is relevant,^4^, ^22^, ^23^ but it illustrates that the dysregulation of the SWI/SNF complex is complex and may involve more than a single subunit.

### 4.3 Expression of SMARCA4 and SMARCE1 is associated with expression of ARID1A

In support of this contention, we found a significant correlation between SMARCA4 and SMARCE1 with ARID1A, respectively, which is another subunit of the SWI/SNF complex.^2^ Previously our group provided evidence of the intratumoral heterogeneity of ARID1A. Furthermore, the loss of ARID1A was associated with an increased expression of PD‐L1, as well as with the EBV and MSI status. Interestingly, any loss of ARID1A expression, regardless of the percentage area of the tumor, was shown to be relevant.^5^, ^24^ Thus, a biologically relevant dysregulation of the SWI/SNF complex could affect subclones of a given tumor, for example, by inactivating mutations of *ARID1A*,^25^ and may involve several members of the SWI/SNF complex making it difficult to unravel correlations with patient survival when SMARCA4 or SMARCE1 are explored individually: the expression of SMARCA4 or SMARCE1 did not correlate with patient survival.

### 4.4 Correlation of SMARCA4 and SMARCE1 with p53

*TP53*, a well‐known tumor suppressor, is frequently mutated in different cancer entities including GC.^26^, ^27^ *TP53* mutation is a rare event in EBV‐positive GCs but often observed in chromosomal instable (CIN) tumors.^26^, ^27^ Similar to the SWI/SNF complex, p53 acts as a transcriptional activator to a complex network of genes, which products are involved in various additional signal transduction pathways.^28^ We found a significant correlation, describing that a decreased expression of SMARCA4 is associated with a low expression of p53 in GC. We could confirm the same association for the expression of SMARCE1 and p53. Thus, dysregulation of the SWI/SNF complex appears to be associated primarily with CIN GCs.

### 4.5 Dysregulation of the SWI/SNF complex inversely correlates with MSI and EBV

An integrative genomic analysis of the Cancer Genome Atlas Research Network proposed a roadmap for patient stratification and trials of targeted therapies by categorizing GC into four subtypes: EBV‐, MSI‐, CIN‐, and genomically‐stable GC.^25^ Interestingly, we found an inverse association of the expression of SMARCA4 and SMARCE1 with MSI, respectively, and a trend of an inverse correlation of the expression of SMARCE1 with EBV positivity. These data lend further support to the hypothesis that the dysregulation of the SWI/SNF complex primarily affects CIN‐GC and not MSI‐ and EBV‐GCs.

### 4.6 Concordance of the expression of SMARCE1 and HER2

Comparing the immunohistochemical stainings of SMARCE1 and HER2 we found a complete concordance of the SMARCE1 and HER2 positive areas, indicating a co‐expression of SMARCE1 with HER2. Regarding the results of Sahlberg et al. this co‐expression could fit to the findings that several genes seem to be associated with HER2 in the growth and survival mechanisms of cancer cells.^29^ On chromosome 17q12‐21, *SMARCE1* is located closely to *ERBB2* and both genes can be co‐amplified in cancer. *ERBB2* encodes for Her2/neu a well‐known oncogene, which is targeted by treatment with trastuzumab and a monoclonal antibody against Her2/neu, mainly in the palliative setting, and shows a high tendency to resistance development.^30^, ^31^ The results of our expression analysis indicated a co‐expression, and subsequent ddPCR confirmed co‐amplification in 3 (75%) of 4 *SMARCE1*‐amplified cases. However, the overall expression of SMARCE1 did not correlate with *SMARCE1* amplification and HER2 status. This indicates that the expression of SMARCE1 is regulated in several ways and not only by amplification.

### 4.7 Examination of the potential tumor biological significance of the SWI/SNF complex

We could not find a correlation with the TNM categories, the OS, or TSS for an increased expression of SMARCA4 or SMARCE1 unlike previous studies indicated.^4^, ^18^ Furthermore, SMARCA4 is discussed to be a tumor suppressor for which a loss‐of‐function is relevant, yet we could not confirm a correlation between a low expression or a complete loss of SMARCA4 and the TNM categories, the OS or TSS.^22^, ^23^ One possible explanation for contradictory findings may be related to differences in cohort sizes, staining protocols, and assessment procedures; for example, Liu et al. studied only 122 patients (vs. 468 patients in our cohort), applied a simplified immunoscoring system (only percentage of positive tumor cells), and did not dichotomize their cohort at the median.^4^ Discrepancies may also indicate a difference between Western and Eastern patients, as the studies showing an influence of a dysregulated expression of the subunits on the prognosis were performed using a cohort of Eastern individuals, while our data refer to a cohort of Western individuals.^4^, ^18^ This supports the approach of differentiation between Asian and non‐Asian GC in the therapy planning, as the ethnic background seems to have an influence on prognosis.^32^ Recent studies suggest that SMARCA4 is involved in an E‐cadherin‐dependent induction of epithelial–mesenchymal transition leading to progressive metastasis.^33^, ^34^ We could not find any correlation between the expression of SMARCA4 and the E‐cadherin status like we could not confirm an association with the TNM categories.

While we did not find a significant correlation between patient prognosis and the expression of SMARCA4 and SMARCE1, respectively, we were able to demonstrate that the dysregulation of more than one subunit of the SWI/SNF complex is associated with the ARID1A‐, p53 and microsatellite status. ARID1A is another subunit of the SWI/SNF complex^2^ and shows the same correlation with the microsatellite status, as shown here for SMARCA4/SMARCE1.^5^, ^22^ This leads to the conjecture that the dysregulation of the SWI/SNF is complex and may affect several different subunits simultaneously making it difficult to find correlations with, for example, patient survival or TNM categories for single members of the complex.

### 4.8 Amplification of SMARCE1

Our data indicate that the expression of SMARCE1 is not only regulated by an amplification of *SMARCE1* in GC. These findings are supported by the expression analysis of SMARCA4 and SMARCE1 showing a concordant expression, while the corresponding gene loci are too far apart to be explained by a combined amplification or loss of both corresponding genes as the only regulation source. The exact mechanisms by which SMARCA4 and SMARCE1 and therefore the SWI/SNF complex are regulated are currently unknown.

## 5 CONCLUSIONS

Summing up, our study confirms the differential and heterogeneous expression of SMARCA4 and SMARCE1 in GC. It correlates with ARID1A and seems to be more commonly dysregulated in CIN GCs, compared with EBV‐positive‐ and MSI‐GC. The dysregulation of the SWI/SNF complex is multifaceted making it difficult to find correlations with clinicopathological patient characteristics and patient survival based on single marker analyses, that is, only SMARCA4 or SMARCE1. In addition, our results show that, in a subset of patients, SMARCE1 is dysregulated by *SMARCE1* amplification. Interestingly, we also found a co‐expression of SMARCE1 and Her2/neu. Future studies should explore the possibility of a synergistic effect of SMARCE1 and Her2/neu overexpression in a subset of patients. Regarding the results of Sahlberg et al.^29^ this co‐expression could be part of the mechanisms in the development of resistances against Trastuzumab, holding potential for an improved precision treatment of GC.

## AUTHOR CONTRIBUTIONS

**Katharina Pries:** Conceptualization (supporting); data curation (equal); formal analysis (lead); investigation (equal); software (equal); validation (equal); visualization (lead); writing – original draft (equal); writing – review and editing (equal). **Sandra Krüger:** Data curation (equal); formal analysis (equal); methodology (lead); resources (equal); validation (equal); writing – original draft (equal). **Steffen Heckl:** Formal analysis (equal); investigation (equal); methodology (equal); validation (equal); writing – original draft (equal). **Hans‐Michael Behrens:** Data curation (equal); formal analysis (equal); methodology (equal); software (lead); validation (equal); writing – original draft (equal). **Christoph Röcken:** Conceptualization (lead); data curation (equal); formal analysis (equal); funding acquisition (lead); investigation (equal); project administration (lead); resources (lead); supervision (lead); writing – original draft (equal); writing – review and editing (equal).

## FUNDING INFORMATION

This research did not receive any specific grant from funding agencies in the public, commercial, or not‐for‐profit sectors.

## CONFLICT OF INTEREST STATEMENT

The authors declare that they have no conflict of interest.

## Supporting information


Table S1.

Table S2.

## Data Availability

All data are included in the manuscript and supplemental tables.
