# Supplementary material for: SMARCA4 and SMARCE1 in gastric cancer: Correlation with ARID1A, and microsatellite stability, and SMARCE1 / ERBB2 co‐amplification
Source: Cancer Med. 2023 Mar 14;12(9):10423–37. doi: 10.1002/cam4.5776 (PMC10225191; doi:10.1002/cam4.5776)
Supplement: Supplementary file 1 — Table S1. [file CAM4-12-10423-s001.pdf]

Supplementary Table 1

			Total	SMARCE1 Staining Intensity 3 present		Dysregulation of SMARCE1 and SMARCA4	
				absent	present	Both Hscore low	Both Hscore high
			n (%)	n (%)	n (%)	n (%)	n (%)
**Total**							
				384 (82.1)	84 (17.9)	148 (31.6)	147 (31.4)
**Gender**	n	p(1)		468	0.265	295	0.476
Female			179 (38.2)	142 (79.3)	37 (20.7)	55 (47.4)	61 (52.6)
Male			289 (61.8)	242 (83.7)	47 (16.3)	93 (52.0)	86 (48.0)
**Age Group**	n	p(1)		468	1.000	295	0.201
< 68 Years			237 (50.6)	194 (81.9)	43 (18.1)	68 (46.3)	79 (53.7)
>= 68 Years			231 (49.4)	190 (82.3)	41 (17.7)	80 (54.1)	68 (45.9)
**Localization**	n	p(1)		466	0.026	294	0.526
Proximal stomach			146 (31.3)	111 (76.0)	35 (24.0)	42 (47.2)	47 (52.8)
Distal stomach			320 (68.7)	272 (85.0)	48 (15.0)	106 (51.7)	99 (48.3)
**Laurén phenotype**	n	p(1)		468	0.023	295	0.210
Intestinal			240 (51.3)	190 (79.2)	50 (20.8)	82 (54.3)	69 (45.7)
Diffuse			148 (31.6)	127 (85.8)	21 (14.2)	39 (42.9)	52 (57.1)
Mixed			31 (6.6)	30 (96.8)	1 (3.2)	8 (40.0)	12 (60.0)
Unclassifiable			49 (10.5)	37 (75.5)	12 (24.5)	19 (57.6)	14 (42.4)
**Grading**	n	p(1)		468	0.570	295	0.266
G1 / G2			110 (23.5)	88 (80.0)	22 (20.0)	38 (56.7)	29 (43.3)
G3 / G4			358 (76.5)	296 (82.7)	62 (17.3)	110 (48.2)	118 (51.8)
**T-category**	n	p(2)		468	0.208	295	0.878
pT1a / pT1b			58 (12.4)	48 (82.8)	10 (17.2)	14 (42.4)	19 (57.6)
pT2			53 (11.3)	42 (79.2)	11 (20.8)	21 (67.7)	10 (32.3)
pT3			185 (39.5)	146 (78.9)	39 (21.1)	59 (47.6)	65 (52.4)
pT4a / pT4b			172 (36.8)	148 (86.0)	24 (14.0)	54 (50.5)	53 (49.5)
**N-category**	n	p(2)		467	0.528	294	0.349
pN0			134 (28.6)	109 (81.3)	25 (18.7)	46 (56.1)	36 (43.9)
pN1			64 (13.7)	46 (71.9)	18 (28.1)	17 (40.5)	25 (59.5)
pN2			84 (18.0)	76 (90.5)	8 (9.5)	31 (56.4)	24 (43.6)
pN3a/b			185 (39.6)	152 (82.2)	33 (17.8)	54 (47.0)	61 (53.0)

**Lymph node ratio**	n	p(1)		467	0.336	294	0.728
Low (<0.189)			227 (48.6)	182 (80.2)	45 (19.8)	73 (51.4)	69 (48.6)
High (≥0.189)			240 (51.4)	201 (83.8)	39 (16.3)	75 (49.3)	77 (50.7)
**M-category**	n	p(1)		468	0.647	295	1.000
pM0			378 (80.8)	308 (81.5)	70 (18.5)	118 (50.0)	118 (50.0)
pM1			90 (19.2)	76 (84.4)	14 (15.6)	30 (50.8)	29 (49.2)
**UICC stage**	n	p(2)		467	0.638	294	0.731
IA / IB			81 (17.3)	67 (82.7)	14 (17.3)	24 (51.1)	23 (48.9)
IIA / IIB			99 (21.2)	79 (79.8)	20 (20.2)	34 (54.0)	29 (46.0)
IIIA / IIIB / IIIC			197 (42.2)	161 (81.7)	36 (18.3)	60 (48.0)	65 (52.0)
IV			90 (19.3)	76 (84.4)	14 (15.6)	30 (50.8)	29 (49.2)
**L-category**	n	p(1)		449	0.271	284	0.075
pL0			219 (48.8)	184 (84.0)	35 (16.0)	78 (55.3)	63 (44.7)
pL1			230 (51.2)	183 (79.6)	47 (20.4)	63 (44.1)	80 (55.9)
**V-category**	n	p(1)		448	0.170	283	0.538
pV0			399 (89.1)	329 (82.5)	70 (17.5)	129 (50.2)	128 (49.8)
pV1			49 (10.9)	36 (73.5)	13 (26.5)	11 (42.3)	15 (57.7)
**R status**	n	p(1)		464	1.000	293	1.000
pR0			406 (87.5)	333 (82.0)	73 (18.0)	124 (50.2)	123 (49.8)
pR1 / pR2			58 (12.5)	48 (82.8)	10 (17.2)	23 (50.0)	23 (50.0)
**HER2 status**	n	p(1)		437	0.038	278	0.835
Negative			401 (91.8)	336 (83.8)	65 (16.2)	131 (51.8)	122 (48.2)
Positive			36 (8.2)	25 (69.4)	11 (30.6)	12 (48.0)	13 (52.0)
**SMARCE1 HScore Median**	n	p(1)					
low Hsocre < 120			232 (49.6)				
high Hscore ≥ 120			236 (50.4)				
**ARID1A any Expression vs Complete Loss**	n	p(1)		415	0.165	258	0.000*
Hsocre = 0 (Complete loss)			45 (10.8)	40 (88.9)	5 (11.1)	29 (90.6)	3 (9.4)
Hscore > 0			370 (89.2)	295 (79.7)	75 (20.3)	92 (40.7)	134 (59.3)
**ARID1A Complete Loss or B/W vs Rest**	n	p(1)		437	0.027	271	0.000*
Rest			370 (84.7)	295 (79.7)	75 (20.3)	92 (40.7)	134 (59.3)
Complete Loss or B/W			67 (15.3)	61 (91.0)	6 (9.0)	37 (82.2)	8 (17.8)
**TP53 HScore Median**	n	p(1)		459	0.116	292	0.010

low (Hscore ≤ 91.5)		230 (50.1)	195 (84.8)	35 (15.2)	91 (56.5)	70 (43.5)
high (Hscore > 91.5)		229 (49.9)	181 (79.0)	48 (21.0)	54 (41.2)	77 (58.8)
**TP53 Mutation**	**n** **p(1)**		107	0.445	71	0.213
wildtype or silent mutation		72 (67.3)	59 (81.9)	13 (18.1)	25 (54.3)	21 (45.7)
mutation		35 (32.7)	26 (74.3)	9 (25.7)	9 (36.0)	16 (64.0)
**SMAD4 Cyt Hscore Q123/Q4**	**n** **p(1)**		458	0.128	292	0.090
Q123		364 (79.5)	305 (83.8)	59 (16.2)	121 (53.1)	107 (46.9)
Q4		94 (20.5)	72 (76.6)	22 (23.4)	26 (40.6)	38 (59.4)
**SMAD4 Nuc Hscore 0**	**n** **p(1)**		458	0.220	292	0.553
present		260 (56.8)	219 (84.2)	41 (15.8)	89 (52.0)	82 (48.0)
absent		198 (43.2)	158 (79.8)	40 (20.2)	58 (47.9)	63 (52.1)
**H. pylori**	**n** **p(1)**		396	0.267	244	0.718
Negative		336 (84.8)	275 (81.8)	61 (18.2)	108 (51.9)	100 (48.1)
Positive		60 (15.2)	53 (88.3)	7 (11.7)	17 (47.2)	19 (52.8)
**EBV status**	**n** **p(1)**		453	1.000	286	0.169
Negative		434 (95.8)	356 (82.0)	78 (18.0)	133 (48.9)	139 (51.1)
Positive		19 (4.2)	16 (84.2)	3 (15.8)	10 (71.4)	4 (28.6)
**MSI status**	**n** **p(1)**		452	0.170	283	0.000*
MSS		417 (92.3)	339 (81.3)	78 (18.7)	116 (46.0)	136 (54.0)
MSI		35 (7.7)	32 (91.4)	3 (8.6)	26 (83.9)	5 (16.1)
**E-Cadherin**	**n** **p(1)**		431	0.062	270	0.675
negative		317 (73.5)	268 (84.5)	49 (15.5)	105 (52.0)	97 (48.0)
positive		114 (26.5)	87 (76.3)	27 (23.7)	33 (48.5)	35 (51.5)
**ß-Catenin**	**n** **p(1)**		433	0.451	271	0.222
negative		245 (56.6)	204 (83.3)	41 (16.7)	80 (53.0)	71 (47.0)
positive		188 (43.4)	151 (80.3)	37 (19.7)	54 (45.0)	66 (55.0)
**Overall Survival [months]**	**p(3)**		456	0.913	288	0.326
Total / events / censored			374/288/86	82/66/16	144/109/35	144/115/29
Median Survival			14.6 ± 1.1	16.0 ± 4.0	16.0 ± 3.3	14.7 ± 1.3
95% C.I.			12.6 - 16.7	8.2 -23.8	9.6 - 22.4	12.1 - 17.3
**Tumor Specific Survival [months]**	**p(3)**		427	0.471	267	0.805
Total / events / censored			349/238/111	78/51/27	132/88/44	135/89/46
Median Survival			15.5 ± 1.4	22.2 ± 2.7	17.9 ± 4.6	15.5 ± 1.5
95% C.I.			12.8 - 18.2	16.9 -27.5	8.8 - 26.9	12.6 - 18.4

[1] Fisher's exact test

[2] Kendall's tau test

[3] log-rank test

* significant after multiple testing correction
