# Supplementary material for: SMARCA4 and SMARCE1 in gastric cancer: Correlation with ARID1A, and microsatellite stability, and SMARCE1 / ERBB2 co‐amplification
Source: Cancer Med. 2023 Mar 14;12(9):10423–37. doi: 10.1002/cam4.5776 (PMC10225191; doi:10.1002/cam4.5776)
Supplement: Supplementary file 2 — Table S2. [file CAM4-12-10423-s002.pdf]

Supplementary Table 2

No.	SMARCE1 HScore	CNV SMARCE1 First ddPCR	CNV SMARCE1 second ddPCR	SMARCE1 Amplification	ERBB2 Amplification	Her2/neu immunohistochemistry	Comment
**SMARCE1 and HER2: complete concordance**							
31	280	8.82	9.6	+	+	2+	
25	255	n.d.	n.d.	n.d.	-	3+	HER2 amplification not assessable.
26	250	10.4	11.40	+	+	3+	
17	230	20.00	19.30	+	+	3+	
18	220	n.d.	n.d.	n.d.	+	3+	
34	215	n.d.	n.d.	n.d.	+	3+	
30	185	n.d.	n.d.	n.d.	+	3+	
32	185	n.d.	n.d.	n.d.	+	2+	
8	175	n.d.	n.d.	n.d.	+	3+	
4	165	n.d.	n.d.	n.d.	+	3+	
5	160	n.d.	n.d.	n.d.	+	3+	
13	160	n.d.	n.d.	n.d.	+	2+	
9	140	n.d.	n.d.	n.d.	+	3+	
24	140	n.d.	n.d.	n.d.	-	3+	HER2 amplification not assessable.
1	130	n.d.	n.d.	n.d.	+	3+	
6	110	n.d.	n.d.	n.d.	+	2+	
27	105	n.d.	n.d.	n.d.	+	3+	
14	95	n.d.	n.d.	n.d.	+	3+	
15	85	n.d.	n.d.	n.d.	+	3+	
2	50	n.d.	n.d.	n.d.	+	3+	
**SMARCE1 and HER2: partial concordance**							
11	230	n.d.	n.d.	n.d.	+	3+	Partial concordance. SMARCE1 and HER2 positive areas match. Tumor shows a strongly SMARCE1 positive area which is HER2 negative.
12	190	2.21	n.d.	-	+	3+	HER2 is heterogeneously expressed. HER2 positive areas are strongly SMARCE1 positive.
33	180	n.d.	n.d.	n.d.	+	3+	HER2 is heterogeneously expressed. SMARCE1 is largely positive but does not show any variation.
7	170	n.d.	n.d.	n.d.	+	3+	Most of the tumor is mucinous. Concordance only in not mucinous part of the tumor. Mucinous part is completely HER2 negative but SMARCE1 positive.
19	115	n.d.	n.d.	n.d.	+	3+	HER2 is heterogeneously expressed. HER2 positive areas are strongly SMARCE1 positive.
28	110	n.d.	n.d.	n.d.	+	2+	Partial concordance. SMARCE1 and HER2 positive areas match. Tumor shows a strongly SMARCE1 positive area which is HER2 negative.
29	85	n.d.	n.d.	n.d.	-	3+	HER2 negative areas partly SMARCE1 positive. HER2 amplification not assessable.
21	75	n.d.	n.d.	n.d.	+	3+	HER2 is heterogeneously expressed. HER2 positive areas are strongly SMARCE1 positive.
**SMARCE1 and HER2: no concordance**							
35	170	n.d.	n.d.	n.d.	-	3+	HER2 positive and SMARCE1 positive areas are separately spread. HER2 amplification not assessable.
16	165	n.d.	n.d.	n.d.	-	3+	Large HER2 positive areas are SMARCE1 negative. HER2 amplification not assessable.

23	120	n.d.	n.d.	n.d.	+	2+	Small HER2 positive area is completely SMARCE1 negative. Large SMARCE1 positive areas are HER2 negative.
22	105	n.d.	n.d.	n.d.	+	3+	Large HER2 positive area missing in SMARCE1 staining. Small HER2 positive area is SMARCE1 negative. Large SMARCE1 positive areas are HER2 negative.
20	70	n.d.	n.d.	n.d.	-	3+	HER2 positive areas are mixed SMARCE1 positive and negative. HER2 amplification not assessable.
36	50	n.d.	n.d.	n.d.	+	3+	SMARCE1 mostly negative (H-score: 50!). Positive areas are separately spread.
3	1	n.d.	n.d.	n.d.	+	3+	Small HER2 positive area is completely SMARCE1 negative (H-score: 1!).
10	/	n.d.	n.d.	n.d.	+	3+	No tissue left for SMARCE1 immunostaining.
37	100	n.d.	n.d.	n.d.	+	3+	HER2 positive area is missing in SMARCE1 stained tissue section
**High SMARCE1 expression (potentially amplified cases)**							
38	230	2.33	2.48	-	-	0	
39	270	3.99	3.81	+	-	0	
40	280	2.08	2.39	-	-	0	
41	230	2.08	2.08	-	-	1+	
42	205	2.56	2.59	-	-	0	
43	220	2.29	2.36	-	-	0	
44	255	2.29	2.51	-	-	0	
45	170	2.77	2.85	-	-	1+	
46	200	2.19	2.20	-	-	1+	
47	220	2.31	2.21	-	-	0	
48	270	1.89	1.94	-	-	0	
49	240	2.06	n.a.	-	-	0	
50	210	2.03	2.27	-	-	0	
**Low SMARCE1 expression (potentially non-amplified cases)**							
51	5	2.42	2.59	-	-	0	
52	5	2.24	2.90	-	-	0	
53	5	2.01	2.37	-	-	0	
54	5	2.23	2.53	-	-	2+	
55	190	2.83	2.83	-	-	0	
56	200	2.28	2.44	-	-	0	
57	197	2.3	2.38	-	-	0	
58	190	2.84	3.14	-	-	0	
59	210	2.35	2.48	-	-	n.d.	
60	191	2.55	2.31	-	-	0	
61	205	2.31	2.24	-	-	0	
62	197	2.26	n.a.	-	-	0	
63	195	2.6	2.57	-	-	0	
64	205	1.91	1.95	-	-	0	
65	195	3	3.30	-	-	0	
66	195	2.55	2.42	-	-	n.d.	
67	195	2.59	2.42	-	-	n.d.	

n.a. = not assessable
n.d. = not data
